# Developing and testing a strategy to enhance a palliative approach and care continuity for people who have dementia: study overview and protocol

**DOI:** 10.1186/1472-684X-11-4

**Published:** 2012-04-02

**Authors:** Christine Toye, Andrew L Robinson, Moyez Jiwa, Sharon Andrews, Fran McInerney, Barbara Horner, Kristi Holloway, Brigit Stratton

**Affiliations:** 1 School of Nursing & Midwifery, Curtin Health Innovation Research Institute, Curtin University, GPO BOX U1987, Perth, WA 6845, Australia; 2 School of Nursing and Midwifery, University of Tasmania, Private Bag 121 Hobart, Tasmania 7001, Australia; 3 Curtin Health Innovation Research Institute, Curtin University, GPO BOX U1987, Perth, WA 6845, Australia; 4 Wicking Dementia Research and Education Centre, University of Tasmania, Private Bag 121 Hobart, Tasmania 7001, Australia; 5 School of Nursing & Midwifery (Victoria), Faculty of Health Sciences, Australian Catholic University, Locked Bag 4115, Fitzroy MDC, Victoria 3065, Australia; 6 Centre for Research on Ageing, Curtin Health Innovation Research Institute, Curtin University, GPO BOX U1987, Perth, WA 6845, Australia; 7 Western Australian Centre for Cancer and Palliative Care, Curtin Health Innovation Research Institute, Curtin University, GPO BOX U1987, Perth, WA 6845, Australia; 8 Wicking Dementia Research and Education Centre, University of Tasmania, Private Bag 121 Hobart, Tasmania 7001, Australia

**Keywords:** Dementia, Palliative care, Evidence translation, Action research

## Abstract

**Background:**

Typically, dementia involves progressive cognitive and functional deterioration, leading to death. A palliative approach recognizes the inevitable health decline, focusing on quality of life. The approach is holistic, proactive, supports the client and the family, and can be provided by the client's usual care team.

In the last months of life, distressing symptoms, support needs, and care transitions may escalate. This project trialed a strategy intended to support a consistent, high quality, palliative approach for people with dementia drawing close to death. The strategy was to implement two communities of practice, drawn primarily from service provider organizations across care sectors, supporting them to address practice change. Communities comprised practitioners and other health professionals with a passionate commitment to dementia palliative care and the capacity to drive practice enhancement within partnering organizations.

Project aims were to document: (i) changes driven by the communities of practice; (ii) changes in staff/practitioner characteristics during the study (knowledge of a palliative approach and dementia; confidence delivering palliative care; views on death and dying, palliative care, and a palliative approach for dementia); (iii) outcomes from perspectives of family carers, care providers, and community of practice members; (iv) the extent to which changes enhanced practice and care continuity; and (v) barriers to and facilitators of successful community of practice implementation.

**Methods/design:**

This action research project was implemented over 14 months in 2010/11 in metropolitan Perth, Western Australia and regional Launceston, Tasmania. Each state based community of practice worked with the researchers to scope existing practice and its outcomes. The research team compiled a report of existing practice recommendations and resources. Findings of these two steps informed community of practice action plans and development of additional resources. Change implementation was recorded and explored in interviews, comparisons being made with practice recommendations. Changes in staff/practitioner characteristics were evaluated using survey data. Findings from semi structured interviews and survey administration established outcomes from perspectives of family carers, care providers, and community of practice members. Consideration of processes and outcomes, across the two state based settings, informed identification of barriers and facilitators. Community of practice reflections also informed study recommendations.

## Background

Dementia is a syndrome resulting from changes in the brain caused by a variety of disease processes [1]. Typically, dementia is progressive and life limiting, with memory and cognition affected and a resultant deterioration in functional status [2]. The dementia 'journey' generally extends over at least several years, with disability gradually increasing. Towards the end of life, total dependency is likely; mobility, understanding, and communication become severely compromised and nutrition and hydration are problematic [3]. Pain may be experienced from concurrent illness/disease or because of immobility (eg, from contractures or pressure ulcers) [2]; agitation is common, with the trigger for this sometimes difficult to determine [4]; and infections are considered inevitable [2].

Globally, numbers of people with dementia are increasing because the prevalence of dementia increases with age and many nations are experiencing population ageing [1]. In Australia, projections indicate an increase from 257,275 people with dementia in 2010, to 1,130,691 people in 2050 [5]. In this country, people with care needs resulting from dementia may be supported to remain at home via the delivery of care packages or they may live in Residential Care Homes (RCHs), known in Australia as Residential Aged Care Facilities. Implementing palliative care is recommended in life limiting conditions in both community based aged care services [6] and RCHs [7]. Data from 2008/9 highlight the importance of these recommendations; in this period, 20% of dementia specific, high level, home care package recipients died [8] and the mean length of stay for those living in RCHs (approximately 60% of whom have a dementia diagnosis) was just 147 weeks, with 88% of separations occurring because of death [9].

Palliative care is recommended for both malignant and non-malignant life limiting disease; it is delivered by a multidisciplinary team and is holistic, aiming to enhance quality of life and death, attempting neither to lengthen or shorten life, and supporting the family [10]. Specialist palliative care services are traditionally tailored to meet the needs of cancer patients [11], but a palliative approach adheres to the same key tenets and can be delivered on a needs basis by the generalist or aged care team throughout any life limiting conditions, including dementia [7]. Support from specialist palliative care is provided when needed [7].

Evidence based guidelines for a palliative approach in community settings [6] and in RCHs [7] were published by the Australian Government in 2011 and 2006 respectively. However, how a high quality palliative approach may be consistently addressed within and across community, residential, and acute care settings is a topic meriting further attention. Our project addressed this issue for people with dementia whose health was deteriorating towards the end of life, and who, therefore, were at particular risk of moving across care sectors (eg, from home to hospital and then into residential care) and receiving care from multiple providers (due to additional service visits or because of transfers). Our strategy was to implement a supported Community of Practice (CoP) drawn from service providers in each of two Australian states, Western Australia and Tasmania. A CoP is defined by Etienne Wenger as a group of clinicians with a common concern, problem, or passion. CoP members can enhance their knowledge and expertise through ongoing interaction, given that learning then becomes a social experience [12]. Instigating CoPs has therefore been promoted as a catalyst for change in facilitating the uptake of evidence among clinicians [13].

Project aims were to document: (i) changes driven by the communities of practice to improve the delivery of a palliative approach for people with dementia drawing close to death; (ii) changes in partner organizations' staff/practitioner characteristics during the study (their knowledge of a palliative approach and dementia; confidence delivering palliative care; views on death and dying, palliative care, and a palliative approach for dementia); (iii) outcomes from perspectives of family carers, care providers, and community of practice members; (iv) the extent to which changes enhanced practice and care continuity; and (v) barriers to and facilitators of successful CoP implementation. The project was undertaken over 14 months, across 2010 and 2011.

## Methods/design

The study employed Action Research (AR), which aims to foster participants' engagement in the research process, facilitating the understanding of issues of those involved in, and affected by, the research [14]. AR proceeds through a helical spiral of planning, action, data collection, analysis, and critical reflection and several cycles may be needed to address the problem or issue under investigation [15]. Change may occur across multiple levels, evidencing as improvements in practice [16]; in this project, improvements were sought in the care of people with dementia drawing close to death. Change may also be demonstrated in enhanced professional relationships and changes in understanding, which, in turn, may lead to changes in the language practitioners use to describe their situations [15]. To create sustainable change, action plans must be appropriate, attainable, and reflect consensus [15]; a core methodological imperative is also to document the process of change [15].

A key strategy of AR is to form an Action Research Group (ARG). By working through the AR spiral, ARG members drive change [14] and the imperative to foster a collaborative ethic within the group is central to facilitating change [17]. Collaboration is evident in shared processes of data collection and analysis, reflection on the data, and action planning and implementation [18]. Collaboration also manifests in shared reflection on action and evaluative processes [18]. As such, involved practitioners take a key role in driving a process of collaborative inquiry [19] because AR aims to place enquiry and decision making processes in the hands of those whose practice is being examined. AR is therefore the preferred method when people want to better understand and improve their situation [17].

In our study, the CoPs in each state constituted the ARGs, which identified problems and drove a change agenda; this was facilitated by the research team, which worked concurrently to build capacity within the CoPs to engage with the AR process. A Reference Group (RG) was also established in each state to advise on and monitor the CoPs' progress in exploring the issues and facilitating change, and to bring additional expertise to the process. Although each CoP operated slightly differently due to the variable practice settings of participants, professional groups involved, and group dynamics, both CoPs operationalized the principles underpinning AR. Both CoPs worked through a series of phases that included mini-cycles of activity, with specific activities varying between the two States. Common phases were: (a) establishing the CoPs and conducting reconnaissance to inform CoP decision making, (b) CoP identification of and reflection upon key practice issues, (c) the CoPs' development and implementation of action plans for practice enhancement, and (d) evaluations informing CoP reflection upon changes made and subsequent outcomes. The AR process does not end abruptly [20] and additional cycles of action were anticipated after the formal end of the project.

### Study populations and settings

Our study's 'target group' comprised people with dementia who were drawing close to death, and who were receiving services from partnering service providers. We used criteria for dementia documented by the American Psychiatric Association [21] and targeted those with advanced dementia as well as those with an earlier stage of dementia plus either another advanced chronic disease known to be life limiting, multiple co morbidities likely to limit life, or frailty (as defined by Fried and colleagues) [22].

The diagnosis of dementia was accepted when this had been documented by a physician or verbally confirmed by health care staff with a physician. Such a diagnosis was also accepted when family or staff reported that the person had dementia, combined with staff evaluation that the person's status fitted within the Functional Assessment Staging Tool's (FAST) stages 3 to 7 [23]. Because cognitive impairment scale scores of the Psychogeriatric Assessment Scales [24] (PAS) are routinely documented for those receiving aged care services in Australia, and high scores are a reliable indicator of dementia [24], we considered these as well. Therefore, a diagnosis of dementia was also accepted when family or staff reported that the person had dementia and the PAS cognitive impairment score was 10 or greater.

People with dementia were considered to be 'drawing close to death' when death in the next 6 months would not be surprising to the clinicians providing their care. Such an approach is consistent with Lynn's recommendations regarding when to address end of life care planning for this population group, given the subtle changes that may signal a final health decline [11].

Because of the health status of our 'target group', we did not aim to collect data from these individuals. Instead, we collected data from the CoP members and from the following groups of those providing care:

1. Current and bereaved family carers (relatives or very close friends of the person with dementia) whom staff would normally contact regarding care related decision making, and who had been involved with care or support at least monthly on average during the previous three months/last three months of the person's life.

2. Professional practitioners, defined as those making care related decisions; these might be nurses, allied health practitioners, physicians, or, in some situations, care managers.

3. Support workers, supervised by professional practitioners.

4. Key informants, drawn from a pool of staff with knowledge and experience of the relevant partner organizations and nominated by RG members.

Settings involved in our study were wherever care was provided to our target group of clients, or support was provided to their family carers, by partnering organizations; they included hospital wards, RCHs, general practitioners' surgeries, support group venues, and clients' own homes. These settings were located within the two, contrasting, local areas: the South Eastern suburbs of metropolitan Perth (Western Australia's capital city of approximately 1.5 million people [25], on this state's West coast) and Launceston (population approximately 71 thousand people [25] in the North of Tasmania, an island state off the coast of South Eastern Australia).

### Protocol

Preliminary work established relationships with partner organizations and addressed issues including project governance, a communication plan, and the roles of RG and CoP members. RG members were drawn from partnering organizations to expand our understanding of these, or they contributed because of their key expertise (in dementia care, palliative care, general practice, pharmacy, or as consumer representatives).

Appropriate approvals were obtained from the Human Research Ethics Committees of Curtin University (HR41/2010), the University of Tasmania (H11217), and the Western Australian South Metropolitan Area Health Service (C/10/246). The research also complied with the World Medical Association's Declaration of Helsinki [26]. All potential participants received detailed information about the study and written informed consent to participation was required before data were collected except when consent was implied by survey completion.

The four phases of the primary AR cycle common to both CoPs are outlined here. Throughout the project, CoP meetings were audiotape recorded with participants' consent. Processes were also logged by the research team to ensure that these could be accurately and comprehensively documented.

#### Phase I: Community of practice recruitment, reconnaissance

CoP participants were recruited on the basis of their passion for implementing a palliative approach in dementia care and their capacity to drive practice enhancement; mainly they were employed by the partnering organizations but some were independent practitioners. Very few CoP members had prior experience of either working in a CoP or engaging with AR. In Western Australia, CoP members were recruited from relevant acute, community, and residential aged care providers, also from general practice; physicians, nurses, and care managers were included. In Tasmania, members were recruited from acute care settings, RCHs, family practices, and community dementia and palliative care organizations; medical, nursing, allied health, and psychology disciplines were included.

In this Phase, CoP members met regularly to consider issues of concern relevant to the project, participating in a series of reconnaissance meetings in which they shared experiences of their practice in caring for people drawing close to death with dementia. During this process, they identified capacity deficits within the CoPs and/or among service providers with respect to knowledge and understandings of dementia and care processes.

Concurrently, the identification of resources to support best practice was undertaken by the research team via an extensive literature and document search. A key aim of the search for practice guidance was to obtain rigorously developed and up to date practice guidelines in the relevant areas, particularly those comprising 'dementia specific' recommendations. From findings, a comprehensive list was compiled of practice recommendations and other resources suitable for supporting the provision of palliative dementia care. Reports of findings were provided to CoP and RG members.

Describing current practice and its outcomes, plus relevant practitioners' characteristics, was a collaborative effort between CoP members and the research team in each State; it was also supported by the RG. This step was crucial to establish a "multi-faceted baseline" [27] to map current practice. Table 1 summarises data collection and analysis for the study. 

**Table 1:** Data collection and analysis

Study objective	Participants	Tools	Phase	Protocol	Analysis
To document changes made.	Key informants from study settings.	Interview schedule and demographic details questionnaire.	I and IV	Volunteers were recruited from staff members nominated by the organization specific RG member because of their knowledge of the organization. Semi structured, audio taped, individual interviews describing current practice and education were conducted; demographic details were obtained at interview and interviews were supplemented with a document audit. Phase I data documented practice at baseline. In Phase IV, we obtained reports of changes implemented during the project.	Responses from interviews and audits were tabulated under category headings. Demographic details were summarized for groups.

To document changes made.	Organized by CoP members.	Logs to record changes in education and practice.	III	Numbers were recorded of staff/family carers/people with dementia included in each change made.	Summary statistics.

To document changes in staff/practitioners' confidence with delivering palliative care; views on death/dying, palliative care, and a palliative approach for dementia.	Professional practitioners in included settings.	Palliative Care Providers' Evaluation Tool, from Eagar and associates' Palliative Care Evaluation Toolkit [28] plus dementia specific questions. Demographic details form.	I and IV	Staff lists were obtained from partner organizations. A coded questionnaire was delivered to each staff member for return into a locked box. The box was emptied by the research team. In this way, we obtained baseline data and post intervention data. This data was entered into a database using IBM's Statistical Package for the Social Sciences (SPSS).	Paired statistical comparisons were undertaken of individual item scores (Phase I versus Phase IV). Demographic details were summarized for groups and facilitated inter-group comparisons.

To document changes in practitioners' knowledge of a palliative approach and dementia.	Professional practitioners attending relevant education sessions in included settings.	Dementia Knowledge Assessment Tool (Version 2)^a^. Palliative Approach Questionnaire^b ^Demographic details (if not already known).	III (data collection) and IV (analysis).	Coded questionnaires were administered and collected by the research team before and after relevant education sessions.	We carried out paired pre-post education statistical comparisons of summed scores and individual item scores. Demographic details were summarized for groups and facilitated inter-group comparisons.

To document outcomes from the perspective of family carers.	Family carers of people drawing close to death with dementia (included settings). Bereaved family carers of people who had died with dementia (also included settings).	Satisfaction With Care at the End of Life in Dementia (SWC-EOLD) scale [29]. Demographic details form. Interview schedule. Questionnaires requesting feedback on practice changes/new resources (Phase IV only).	I - current and bereaved carers. IV - current carers.	Recruitment was via a letter of invitation sent out by the staff of the partnering organization. Interviews were generally face to face but some were conducted over the telephone. Interviews addressed practice and associated outcomes for clients and families; all were audiotape recorded and transcribed. The SWC-EOLD [29] was administered during the interview. Phase I obtained baseline data. In Phase IV, obtaining post intervention data, the letter of invitation was accompanied by a feedback questionnaire and demographic details form. Feedback data and accompanying demographic data were entered into an SPSS database.	Using QSR International's NVivo software to support categorization of qualitative data, thematic analysis was carried out by the interviewer and an experienced researcher. Demographic details and SWC-EOLD data were incorporated in reports of qualitative findings. Feedback and accompanying demographic data were summarized using descriptive statistics. Comments were also summarized.

To document outcomes from the perspective of care providers.	Professional practitioners and support workers (included settings)	Feedback questionnaires and items.	III and IV	Feedback questionnaires were provided for all staff attending education sessions. Items were also added to the Phase IV survey of professional practitioners requesting feedback on changed protocols and new resources.	We calculated descriptive statistics and any comments were summarized.

To document outcomes from the perspective of care providers.	Key informants (included settings).	Interview schedule.	I and IV	Key informant interviews included questions addressing outcomes of practice at baseline (Phase I) and after change was implemented (Phase IV).	Relevant responses from interviews were categorized.

To document outcomes from the perspective of CoP members.	All consenting CoP members.	Interview schedule. Demographic details form. Capacity Building Checklist from Eagar and associates' Palliative Care Evaluation Toolkit [28].	IV	Recruitment was via a letter of invitation sent out by the research team. Interviews were individual and face to face. The checklist was completed at the start of the interview. Thereafter, all interviews were audiotape recorded and transcribed. Data from the checklist were entered in to an SPSS database.	Using the NVivo software program to support categorization of data, thematic analysis was carried out by the interviewer and an experienced researcher. This analysis provided descriptions of CoP members' perceptions of processes and their outcomes. Checklist data were summarized using descriptive statistics.

To document the extent to which changes enhanced practice and care continuity.	Research team.	Reports detailing practice recommendations (Phase I), practice at baseline and its outcomes (Phase I), and changed practice and outcomes (Phase IV).	IV	Overall reporting by the research team examined changes in education, practice, and outcomes, comparing practice changes across sectors and with practice recommendations.	Enhanced practice was viewed as that which aligned more closely with practice recommendations and/or led to improved outcomes. A more consistent approach across sectors that aligned with practice recommendations was regarded as enhancing care continuity.

To document barriers to/facilitators of CoP implementation.	Research team and CoP members.	Process log and audiotape recordings of CoP meetings. CoP interview scheduling also facilitated critical reflection by CoP members.	All.	Processes were logged throughout the project by the project officers in the two states. Logging included recording each CoP meeting's aim and outcomes, along with field notes. With the consent of CoP members, CoP meetings also were audiotape recorded. CoP interviews included reference to barriers and facilitators.	A table of barriers to and facilitators of CoP implementation was constructed from these data. A summary of process and contextual differences between the project's implementation in each state and subsequent outcomes was also synthesized by the research team and investigators; this informed an overall description of barriers and facilitators.

In this Phase, as shown in Table we collected data across the relevant partner organization practice settings from:

• key informants, supplemented by a document audit, to obtain a description of current practice and its outcomes;

• professional practitioners, to ascertain their confidence in delivering palliative care and their views on death and dying, palliative care, and palliative care for dementia; and

• current and bereaved family carers, to obtain their perspectives of care and support and its outcomes.

Reports were again provided to the CoP members and RG members/partner organizations. These reports included a brief review of the extent to which practice was consistent with the previously identified best practice recommendations. Comparisons of findings among organizations in the same local areas facilitated understandings of care continuity. The reconnaissance therefore informed the CoPs' reflections upon the needs for change.

#### Phase II: Identifying and reflecting upon the issues

Over subsequent meetings, CoP members in each State critically reflected upon the reports provided in Phase I and refined their understandings of key issues of concern identified during the reconnaissance meetings. Collaborative engagement with the research team grew, ensuring that CoP members' knowledge and expertise were central to identifying potential areas of action and change. Consistent with AR, such an approach also facilitated ownership of the data and change processes.

Methods used to facilitate engagement varied between the two CoPs. Meetings in Perth were facilitated using strategies to promote rapid decision making dominated by passionate commitment. Strategies in Launceston emphasized a more critically reflective and information seeking approach. In both States, this step generated a list of 'issues of concern' for consideration as areas in which actions might be undertaken during the project.

#### Phase III: Planning and taking action

From their involvement in the Phase II activities, the CoP members' knowledge of practice, capacity, and related outcomes informed their plans for change (action plans) and led them to identify the resources they would need to implement these plans. Resources were developed or modified from existing sources in a collaborative effort that involved extensive input from the Perth based CoP and research team members in particular. CoP members in each State also liaised with relevant staff in the partner organizations to enlist their support.

Facilitated meetings were held monthly to support the CoPs as they implemented their action plans in an effort to bring about the desired changes in practice, using the resources that had been developed. In Perth, CoP members liaised with colleagues in the workplace to form new, local, organizational CoPs that would champion change. In Launceston, based upon their areas of expertise, CoP members identified and then formed partnerships with relevant service providers in sub groups charged with responsibility to refine and implement action plans. Changes in education were also addressed, with knowledge change measured and feedback obtained; logging of changes in both practice and education also occurred throughout this time period (see Table 1).

#### Phase IV: Evaluation and critical reflection

At the end of the project, as shown in Table 1, final evaluations of changes made and their outcomes were undertaken by members of the research team in collaboration with CoP members. Consistent with the baseline evaluations undertaken in Phase I, we collected data across settings from:

• key informants, to obtain organizational perspectives of changed practice and its outcomes;

• professional practitioners, to ascertain changes in confidence and views plus their ratings of the new practices, education, and resources; and

• family carers, to obtain perspectives of care, support, and outcomes, with particular reference to the newly introduced practices and resources.

We also interviewed CoP members to ascertain their perspectives, including their views of changes in organizational capacity brought about by the project (again shown in Table 1). Reports summarizing findings for individual organizations (educational and practice changes achieved and their outcomes) were presented to CoP members, RG members, and partner organizations.

The overall project report also addressed the extent to which practice changes implemented enhanced care and care continuity and barriers to and facilitators of successful CoP implementation. Critical reflection upon the project and its outcomes by CoP members helped to inform the discussion included in this report. Recommendations addressed any need for additional cycles of the research process and for ongoing practice, the establishment of CoPs in future practice change initiatives, and further research in this area.

### Summary

This project was conducted in two, contrasting, geographical locations and an AR methodology was employed when implementing a strategy designed to enhance care for people drawing close to death with dementia. In each location, a CoP was recruited that included members drawn from across care sectors and disciplines; each CoP then identified and drove changes aimed at enhancing a palliative approach for this population group. Changes in education, practice, and outcomes were evaluated; facilitators of and barriers to successful CoP implementation were also assessed. Future papers will provide details of all the actions planned and implemented by each CoP.

## Competing interests

The authors declare that they have no competing interests.

## Authors' contributions

CT and AR contributed to the conception and design of the study, acquisition of data, analysis and interpretation of data, and drafting and revision of the manuscript. MJ and SA contributed to the conception and design of the study, the acquisition of data, and drafting and revision of the manuscript. FM and KH contributed to the conception and design of the study and drafting and revision of the manuscript. BS and BH contributed to the acquisition of data and drafting and revision of the manuscript. All authors read and approved the final manuscript.

## Authors' information

CT was involved in the development of the *Guidelines for a palliative approach in residential aged care *[7] and the *Guidelines for a palliative approach for aged care in the community setting *[6]. MJ is a practicing General Practitioner. KH coordinates a regional community aged care program that includes care provision for people with dementia. SA holds a National Health and Medical Research Council 'Translating Research into Practice' Fellowship.

## Pre-publication history

The pre-publication history for this paper can be accessed here:

http://www.biomedcentral.com/1472-684X/11/4/prepub

## Endnotes

The tools we used to measure changes in knowledge in health care professionals were:

a). The Dementia Knowledge Assessment Tool (Version 2), modified by Toye, Lester, Popescu, McInerney, Andrews, and Robinson in 2009 from the Dementia Knowledge Assessment Tool, a questionnaire developed with funding from the Australian Government to evaluate the (similarly funded) Dementia Care Essentials education program.

b). The Palliative Approach Questionnaire, developed by Andrews, Toye, McInerney, Leggett, and Robinson in 2010 from a tool designed for the doctoral work of the first author conducted in the University of Tasmania's School of Nursing and Midwifery; "*Behind the Blue Door": Developing the practices of aged care staff around a palliative approach in a dementia special care unit*.
